# IgE autoreactivity in bullous pemphigoid: eosinophils and mast cells as major targets of pathogenic immune reactants†

**DOI:** 10.1111/bjd.15924

**Published:** 2017-11-28

**Authors:** P.C. Freire, C.H. Muñoz, G. Stingl

**Affiliations:** ^1^ Department of Dermatology Division of Immunology, Allergy and Infectious Diseases Medical University of Vienna Vienna 1090 Austria

## Abstract

**Background:**

Bullous pemphigoid (BP) is an autoimmune disease characterized by tense blisters that are usually preceded by urticarial eruptions. Affected patients exhibit IgG and/or IgE autoantibodies against BP180 and/or BP230. Their relative importance in disease pathogenesis has not been fully elucidated.

**Objectives:**

The aim of this study was to provide a better characterization of the circulating and tissue‐resident IgE in patients with BP at the serological, structural and functional levels.

**Methods:**

Sera (*n* = 19) and skin (*n* = 33) from patients with BP were analysed via enzyme‐linked immunosorbent assay (ELISA) and immunofluorescence, respectively.

**Results:**

The results obtained show that many patients with BP exhibit elevated IgE levels in the serum and in the skin. In the skin, it is very rarely and only sparsely found along the basement membrane zone, but is prominently present on mast cells and eosinophils. At least a portion of these IgE antibodies are BP‐specific, as evidenced by serum ELISA and by the colocalization of BP180 and FcεRI‐bound IgE on mast cells and/or eosinophils. An important role of these immune reactants can be inferred from our additional finding that cross‐linking of IgE, derived from BP sera, on FcεRI‐expressing rat basophils with BP180 results in robust degranulation of these cells.

**Conclusions:**

We propose the existence of a disease pathway alternative to IgG and complement that may well be responsible for some of the clinical features of this autoimmune disease.

## Abstract 2

**What's already known about this topic?**

- Patients with bullous pemphigoid (BP) very often present with pruritus and hives.
- Circulating disease‐specific IgE is characteristic of patients with BP and correlates with urticarial lesions.

**What does this study add?**

- This study demonstrates that IgE is found not only in the serum, but also in the perilesional skin of patients with BP, colocalizing with mast cells, eosinophils and BP180 fragments.
- Antigenic peptides of BP180 are capable of cross‐linking FcεRI‐bound IgE, ultimately resulting in degranulation.

**What is the translational message?**

- IgE‐mediated events likely influence the onset and development of BP symptoms.
- Uncovering an IgE‐dependent disease pathway might impact therapeutic options and reduce the burden of steroids in this aged population.

Linked Comment: Ujiie. *Br J Dermatol* 2017; **177**:1481–1482.

Plain language summary available online

Respond to this article

Bullous pemphigoid (BP) is a disease that typically occurs in elderly patients, clinically characterized by tense blisters that may follow an urticarial or erythematous phase. The detection of self‐reactive IgG in 1967^1^ placed this disease into the autoimmune category. Typically, direct immunofluorescence (DIF) staining of perilesional skin reveals a linear pattern of IgG and complement factor C3 along the basement membrane zone (BMZ).^2^ Circulating IgG of patients with BP recognize moieties of about 230 kDa (BP230) and 180 kDa (BP180).^3^ Both BP antigens are part of the hemidesmosome, the critical structure for dermoepidermal adhesion. The epitope most commonly identified as the main target for autoantibodies is the noncollagenous domain NC16A of BP180.^4^ Nonetheless, some patients do not (or do not only) show reactivity against this molecular stretch, but rather exhibit other immunodominant epitopes.^5^, ^6^

Despite IgG and complement being hallmarks of BP, their sole action does not easily explain all the characteristics of the disease. For example, animal models using BP180‐specific IgG are characterized by a neutrophilic infiltrate,^7^ whereas eosinophils represent the main cell type found in the human form of the disease.^8^, ^9^ Furthermore, α‐NC16A IgG correlates with blister scores rather than with the extent of inflammation,^10^ and C3 deposition along the BMZ by DIF is not found in approximately 10% of patients with BP.^11^

In 1974, it was first reported that patients with BP display elevated IgE in the serum.^12^ Later, it was demonstrated that some of these circulating IgE autoantibodies are directed against BP antigens.^13^, ^14^ Some authors also observed a correlation between α‐BP180 IgE and disease severity.^15^, ^16^ Furthermore, IgE‐based *in vivo* models of BP supposedly mimic the human disease more closely – with both blister formation and eosinophilic infiltrates – than those dependent on IgG and complement.^17^, ^18^

The location of IgE in BP skin has been a matter of controversy, with positive staining for IgE deposits being described along the BMZ^19^, ^20^, ^21^ or on cells within the inflammatory infiltrate.^22^, ^23^ Despite numerous speculations, the pathogenic significance of these IgE deposits, if any, is far from being clear.

In this study, we wanted to address this issue by taking a closer look at these BP‐specific IgE autoantibodies, investigating their antigenic specificity, cell and tissue distribution, cellular and molecular partners, as well as their functionality.

## Materials and methods

### Sera and skin samples

Sera from patients with BP (*n* = 19) and healthy donors (*n* = 18) were collected from subjects who voluntarily signed an informed consent form. Skin biopsies from patients with BP (*n* = 33) and those with non‐BP skin conditions (*n* = 8) were collected for routine diagnostic purposes. Non‐BP controls comprised patients diagnosed with linear IgA disease (*n* = 1), vasculitis (*n* = 1), urticaria (*n* = 1), epidermolysis bullosa acquisita (*n* = 2), pemphigus vulgaris (*n* = 2) and dermatitis herpetiformis (*n* = 1). Normal human skin (*n* = 5) was obtained from patients undergoing abdominoplastic surgery.

This study was approved by the ethics committee of the Medical University of Vienna (EK1645/2014) and conducted in accordance with the Declaration of Helsinki.

### α‐BP180 and α‐BP230 IgE enzyme‐linked immunosorbent assays

NC16A and BP230 peptide‐covered enzyme‐linked immunosorbent assay (ELISA) plates were obtained commercially (Mesacup, MBL, Nagoya, Japan) and adapted for IgE detection. Details of the detection antibodies used can be found in Data S1 (also see Fig. S1; see Supporting Information).

### Uncovering of the major BP‐180 epitopes

Overlapping peptides spanning the NC16A and C‐terminal domains of BP180 (for peptide sequences see Data S1; see Supporting Information) were kindly provided by Marwa Mostageer and Oskar Smrzka at Affiris AG (Vienna, Austria). IgG and IgE reactivity to each of the peptides was detected via ELISA. An FcεRI fragment was used as an irrelevant peptide and any signal twofold higher than that of the negative control was considered positive.

### Stainings, quantification and stripping of skin‐bound IgE

For immunofluorescence, 5‐μm sections of cryopreserved biopsies were used. Detailed information on the commercial antibodies used is provided in Data S1 (see Supporting Information). Mouse α‐linear IgA disease antigen (LAD)‐1, targeting the 120 kDa fragment of BP180,^24^ was a generous gift from Peter Marinkovich at the Stanford University School of Medicine (Stanford, CA, U.S.A.) and α‐FcεRI blocking antibody (15_1)^25^ was kindly provided by Jean‐Pierre Kinet from Harvard Medical School (Boston, MA, U.S.A.). IgE stripping was performed using a glycine buffer, as previously described.^26^ Quantification was performed with TissueQuest software (TissueGnostics, Vienna, Austria).

### IgE‐mediated basophil degranulation

Human FcεRI‐expressing rat basophil leukaemia cells were a gift from Ryosuke Nakamura (National Institute of Health Sciences, Tokyo, Japan)^27^ and Rudolf Valenta (Medical University of Vienna, Vienna, Austria).^28^ Assays were performed as previously described^29^ (detailed information is provided in Data S1; see Supporting Information). Results were expressed as a percentage of total β‐hexosaminidase release, obtained by lysing the cells with 10 % Triton‐X‐100 (Sigma‐Aldrich Co., St Louis, MO, U.S.A.).

### Graphics and statistical software

Graphics and statistical analyses were performed using Prism 5 (GraphPad, La Jolla, CA, U.S.A.). Student's *t*‐tests were employed for comparing means between two groups at a time. A *P*‐value of < 0·05 was considered statistically significant.

## Results

### Circulating bullous pemphigoid‐specific IgE is elevated in patients with bullous pemphigoid and reacts with the same dominant epitopes as IgG

**Figure 1:**
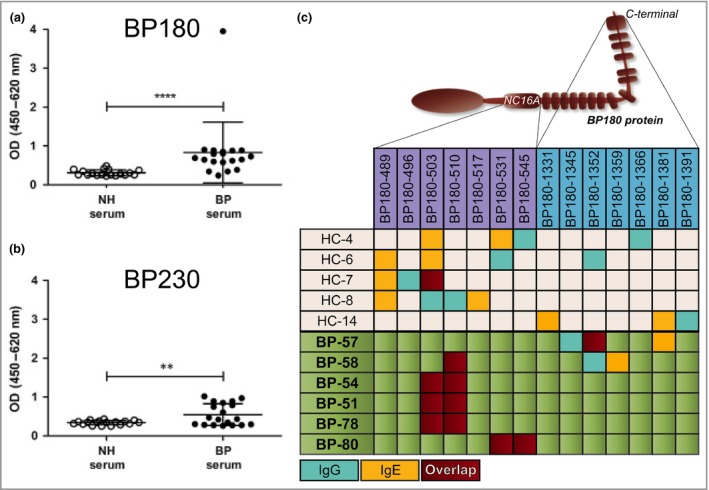
Circulating bullous pemphigoid (BP)‐specific IgE is elevated in patients with BP and reacts with the same dominant epitopes as IgG. (a) α‐BP180 and (b) α‐BP230 IgE antibodies were measured in the peripheral blood of HCs (*n* = 18) or patients with BP (*n* = 19). (c) Normal and BP circulating IgG (blue) and IgE (yellow) were evaluated for reactivity against peptide stretches of BP180, spanning the NC16A or C‐terminal regions. An overlap in IgG and IgE reactivity towards the same peptide stretch is shown in red. The data represented in the graphs are mean ± SD. **P < 0·01, ****P < 0·0001. OD, optical density; NH, normal human; HC, healthy control.

In line with previous literature, we found that patients with BP have significantly higher levels of circulating anti‐BP180 and anti‐BP230 IgE than healthy controls (HCs) (Fig. 1a, b and Fig. S2; see Supporting Information). Consistent with the low concentration in serum, IgE signals in the BP ELISAs were low in all but one patient with BP (BP‐51), who presented with extremely high levels of anti‐BP180 IgE (Fig. 1a, outlier).

To uncover the dominant epitopes for both IgG and IgE and gain a general understanding of how these specific immunoglobulins relate to each other, ELISAs were performed using short peptide sequences of the NC16A and C‐terminal regions of BP180. The results obtained showed that, in patients with BP, both IgG and IgE α‐BP180 antibodies recognized a particular stretch of NC16A in an overlapping fashion (Fig. 1c). Such a BP‐specific motif was not seen in the sera of HC, one‐third of which also contained low levels of α‐BP180 antibodies of the IgG and IgE classes. As shown in Figure 1c, the antibodies from HC seem to recognize different portions of the molecule at random, without any apparent pattern or overlap between the two immunoglobulin classes. Although these results are strongly suggestive of a competition phenomenon between IgG and IgE from patients with BP, we were not able to definitively demonstrate via ELISA that preincubation with purified BP IgG leads to a statistically significant reduction of the specific IgE signal (Fig. S3; see Supporting Information). However, it should be noted that the α‐BP180 IgE signal tended to be lower when the sera incubation was preceded by a blocking step with α‐BP180 IgG vs. nonspecific BP IgG.

### IgE is found in the perilesional skin of patients with bullous pemphigoid, mostly bound to mast cells and eosinophils

**Figure 2:**
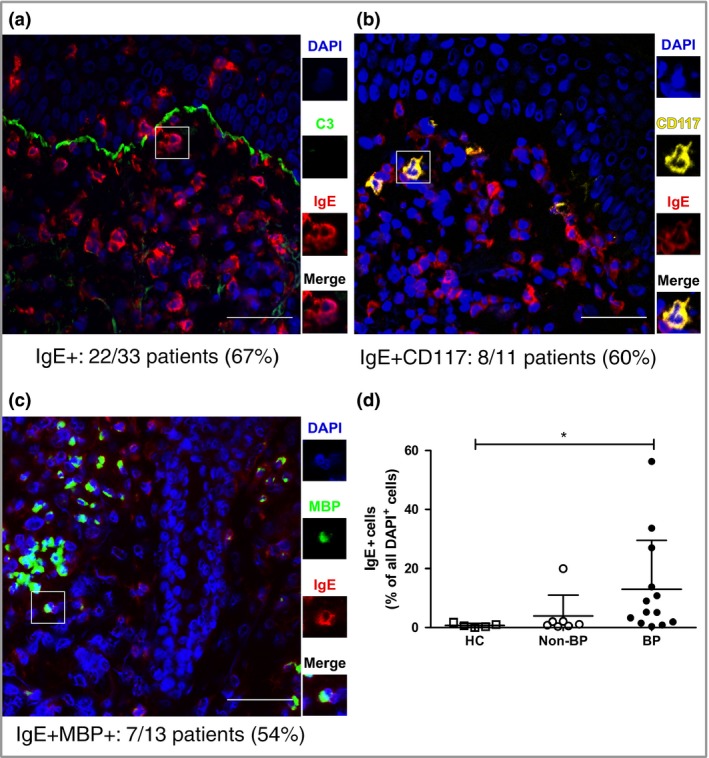
IgE is found in the perilesional skin of patients with BP, mostly bound to mast cells and eosinophils. Direct immunofluorescence of BP perilesional skin for (a) IgE, (b) mast cells (CD117+ ) and (c) eosinophils (MBP+). The number of patients tested and found positive for each case is indicated. Scale bars = 50 μm. (d) IgE+ cells were calculated for healthy controls (HCs) (*n* = 5), non‐BP disease controls (*n* = 7) and patients with BP (*n* = 13). Non‐BP disease controls include linear IgA disease, vasculitis, epidermolysis bullosa acquisita, pemphigus and dermatitis herpetiformis. Positive cells are presented as a percentage of all dermal cells identified by 4′,6‐diamidino‐2‐phenylindole (DAPI) staining. The data represented in the graph are mean ± SD. *P < 0·05. MBP, major basic protein.

By DIF, a large percentage of patients with BP (22 of 33) presented with IgE+ cells in perilesional skin (Fig. 2a). By contrast, skin‐associated IgE was not detected in any of the HCs (Fig. 2d). The strength of the IgE signal and the density of IgE+ cells in BP skin varied largely from patient to patient (Fig. 2d), with no apparent correlation with total or specific serum IgE (Fig. S4; see Supporting Information). Surprisingly, IgE was not found along the BMZ, with the exception of one BP patient (BP‐60) (Fig. S5; see Supporting Information). This patient exhibited a low IgE signal along the BMZ and a stronger signal around cells in the dermis. Generally, IgE‐bearing cells were found to be CD117+ mast cells (Fig. 2b) or major basic protein (MBP)+ eosinophils (Fig. 2c). While mast cells naturally express FcεRI, eosinophils are known to express it only in pathological situations.^30^, ^31^ Similar to others,^32^ we have found MBP and FcεRI colocalizing in BP biopsies (Fig. S6; see Supporting Information), revealing the existence of FcεRI+ eosinophils in these patients. Occasionally, we also observed IgE+ dendritic cells (Freire *et al*., preliminary data), which are likewise known to express FcεRI.^25^ While there were highly significant differences between the eosinophilic infiltrates of HCs and BP, no major changes were found in the percentage of mast cells and eosinophils in BP and other inflammatory skin diseases, such as epidermolysis bullosa acquisita and dermatitis herpetiformis (Fig. S7; see Supporting Information). However, there was a clear tendency towards higher percentages of IgE+ cells in the BP group than in the non‐BP‐diseases group, and a significant increase when compared with HCs (Fig. 2d).

All patients with BP who were tested during this study, regardless of the presence or absence of skin‐bound IgE, had linear deposits of IgG and C3 along the BMZ by DIF. Notably, while circulating BP‐specific IgG subclasses were balanced between IgG1, IgG3 and IgG4, with only a slight dominance of the latter, BMZ‐bound IgG in the skin was predominantly of the IgG4 subclass (Fig. S8; see Supporting Information).

### IgE–cell interaction within the skin is most likely mediated by FcεRI expressed on mast cells and eosinophils

**Figure 3:**
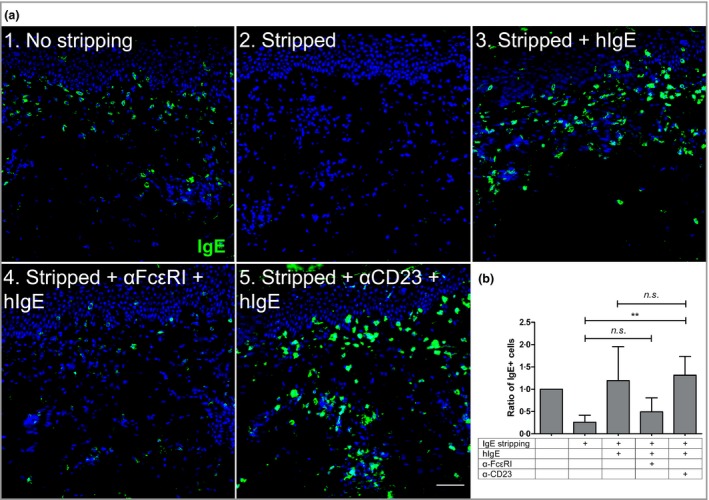
IgE–cell interaction within the skin is most likely mediated by FcεRI expressed on mast cells and eosinophils. (a) Skin‐bound IgE found in perilesional bullous pemphigoid skin (1) was stripped with an acid solution (2) and the sections loaded with human IgE (hIgE) alone (3) or blocked with α‐FcεRI (4) or α‐CD23 (5) antibodies before incubation with hIgE. Scale bar = 50 μm. (b) IgE+ cells were counted for each case and expressed as a function of the original amount of IgE found in the tissue prestripping (*n* = 4). The data represented in the graph are mean ± SD. **P < 0·01. *n.s*., not significant.

In order to unravel the biologically relevant IgE‐binding structure in BP skin, perilesional skin sections from patients who were IgE+ were stripped of skin‐bound IgE as a first step (Fig. 3). This stripping procedure did not affect the capacity of IgE receptors to continue to bind IgE, as proven by the recovery of the IgE signal upon incubation of the stripped sections with human IgE. Our additional finding that blockade of FcεRI, but not of FcεRII/CD23, led to a reduction, on both mast cells and eosinophils, of the IgE signal to poststripping levels, strongly suggests that FcεRI is the critical IgE‐binding moiety in the skin of patients with BP.

### Shed fragments of BP180 are found in the dermis, colocalizing with IgE

**Figure 4:**
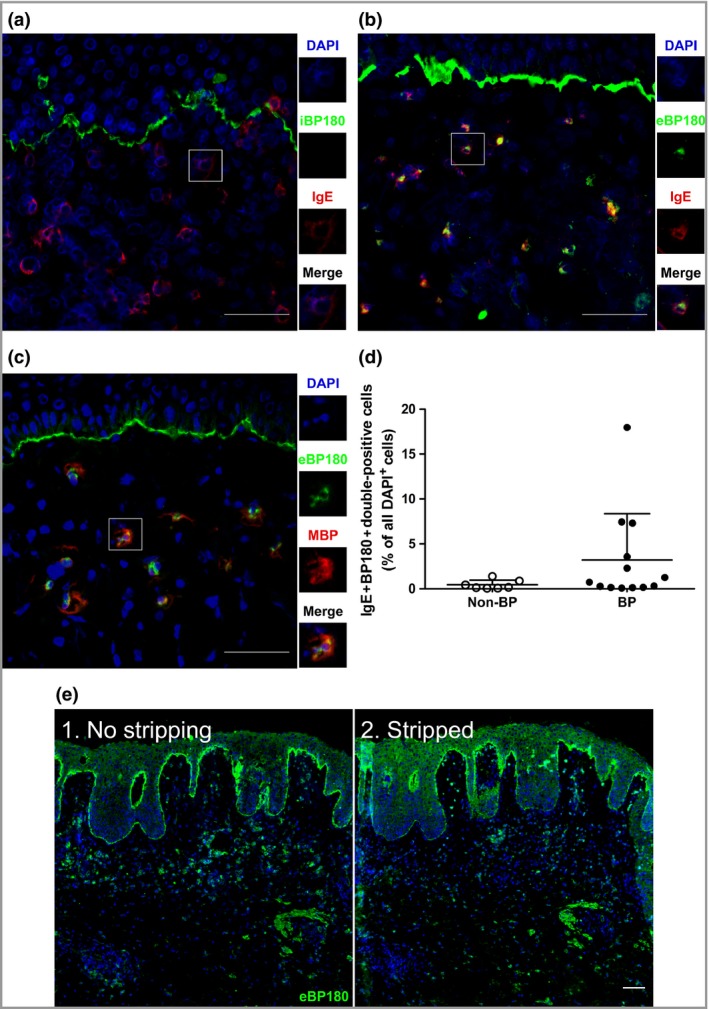
Shed fragments of BP180 are found in the dermis, colocalizing with IgE. Perilesional sections of skin from patients with bullous pemphigoid (BP) were stained for (a, b) IgE, (a) the intracellular moiety of BP180, (b, c) the extracellular moiety of BP180 and (c) major basic protein (MBP) (eosinophils). (d) The percentage of double‐positive (IgE+BP180+) cells, compared with all 4′,6‐diamidino‐2‐phenylindole (DAPI)+ dermal cells, in the skin of BP (*n* = 13) and non‐BP disease controls (*n* = 7), as stained by direct immunofluorescence (DIF). Non‐BP disease controls include linear IgA disease, vasculitis, epidermolysis bullosa acquisita, pemphigus and dermatitis herpetiformis. The data represented in the graph are mean ± SD. (e) DIF for extracellular BP180 moieties (eBP180) in a (1) nonstripped or (2) acid‐stripped BP section (*n* = 1). Scale bars = 50 μm.

Our findings thus far suggest that pathogenic IgE acts via inflammatory cells located in the dermis, rather than via its binding to the BMZ‐associated BP antigens. Although BP180 is mostly found as a transmembrane protein expressed by basal keratinocytes, cleaved forms of its extracellular domain are known to react with BP serum^33^ and can appear in both epidermal and dermal extracts.^34^ When BP180 was stained with an antibody targeting its intracellular domain, a linear signal was found along the BMZ, while the dermis remained clear (Fig. 4a). On the other hand, when the antibody used had the extracellular portion of BP180 as its target, fragments of this protein were found dotting the dermis in addition to the expected BMZ signal (Fig. 4b, c and Fig. S9; see Supporting Information). These dermal dots do not appear to be specific for BP, given that they may also be found in both healthy and non‐BP disease controls (Figs. S7c, S9c; see Supporting Information). As illustrated in Figure S9c (see Supporting Information), this BP180+ material from healthy skin was not clustered at a specific region, but rather appeared to be distributed at random. In patients with BP, by contrast, these dermal BP180 dots were frequently seen in close proximity to, or associated with, IgE+ cells (Fig. 4b, c). In fact, IgE+BP180+ (double‐positive) cells tend to be characteristic of a subgroup of patients with BP, as a result of the higher levels of skin‐bound IgE (Figs 2d, 4d). Based on these data, we have reason to assume that IgE and BP180 can be complexed on the very same cells. It is noteworthy that acidic conditions, which lead to the stripping of cell‐bound IgE (Fig. 3), resulted in an almost complete disappearance of the cell‐bound BP180 signal, but resulted in only a slight reduction of the BMZ‐associated α‐BP180 staining (Fig. 4e).

### IgE‐BP180 complexes are capable of inducing the degranulation of basophils

**Figure 5:**
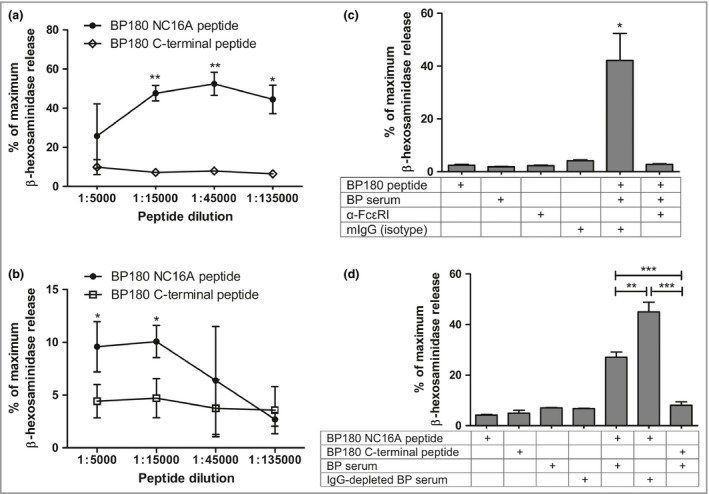
IgE‐BP180 complexes are capable of inducing the degranulation of basophils. FcεRI‐expressing rat basophils were loaded with IgE present in the sera of two patients with BP, (a) BP‐51 and (b) BP‐80, and stimulated with increasing dilutions of highly (closed symbols) or poorly antigenic (open symbols) BP180 peptides (see Fig. 1c). (c) Receptor loading with IgE from patient BP‐51 was preceded by incubation with α‐FcεRI or isotype control. (d) IgG‐depleted or nondepleted BP‐51 sera were tested for their potential to cause basophils to degranulate. Results are expressed as a percentage of maximum β‐hexosaminidase release, achieved by solubilizing cell membranes. The data represented in the graphs are mean ± SD. *P < 0·05, **P < 0·01, ***P < 0·001.

In order to examine the functionality of cell‐bound IgE and BP180 moieties, we have performed an indirect degranulation assay, using rat basophils expressing human FcεRI.^29^, ^35^ As shown in Figure 5, we found that sequential incubation of these cells with BP serum and the BSA‐bound matching BP180 epitope (Fig. 1c) triggered a powerful degranulation event in one sample (BP‐51) and a weak but distinct effect in another one (BP‐80) (Fig. 5a, b). Conversely, BP180 C‐terminal epitopes, for which patients with BP had no reactivity, did not induce specific basophil degranulation. Patients BP‐51 and BP‐80 were found to have the highest levels of specific circulating IgE in our cohort (not shown, data from Fig. 1c). Other patients with BP, in addition to HCs, who were tested against the main NC16A epitope (BP180–510, Fig. 1c), failed to trigger specific basophil degranulation (Fig. S10; see Supporting Information). In agreement with our previous findings, degranulation induced by IgE‐BP180 complexes was dependent on the high‐affinity IgE receptor, as FcεRI blockade prevented the release of β‐hexosaminidase (Fig. 5c). Consistent with our finding that α‐BP180 IgG and IgE compete for the same epitopes, we were able to demonstrate that IgG‐depleted serum induces a significantly stronger mediator release than its nondepleted counterpart (Fig. 5d).

## Discussion

During the past few decades, evidence has emerged that IgE antibodies are not solely directed against exogenous antigens but can also target self‐proteins. Indeed, IgE‐dependent autoimmune phenomena have been described in atopic dermatitis,^36^ lupus^37^ and BP.^20^ In this study, we investigated the characteristics of the IgE response in patients with BP in depth and came to the following conclusions: (i) circulating IgE is not only elevated, but also specific for both BP antigens, BP180 and BP230; (ii) self‐reactive IgE and IgG recognize the same epitopes in the BP180 molecule; (iii) in the skin of patients with BP, IgE was associated with inflammatory cells, such as mast cells and eosinophils, with FcεRI being the major binding structure; (iv) shed fragments of BP180 are found colocalizing with IgE and eosinophils and may be stripped together with IgE; and finally, (v) IgE‐BP180 complexes have the potential to cross‐link FcεRI and cause the degranulation of basophils.

Our finding of elevated total and specific circulating IgE in patients with BP is in agreement with the results of several groups of other investigators.^13^, ^38^, ^39^, ^40^ In contradiction to some investigations,^41^, ^42^, ^43^ we could not reliably detect IgE deposited along the BMZ in either indirect immunofluorescence or DIF, with the exception of one patient (BP‐60). The reason for this discrepancy is not entirely clear. A comparison of our staining method with that of others makes a sensitivity issue unlikely. Given the substantially different concentrations of IgG and IgE in circulation, our experiment that demonstrated a clear overlap between dominant epitopes recognized by these two immunoglobulins provides a good explanation for a lack of detectable α‐IgE staining along the BMZ. Following this reasoning, a positive signal for IgE along the BMZ would only be possible with high IgE and concomitantly low IgG levels. In this regard, our patient cohort may have been different from those of other studies. Interestingly, though, we did not find an abnormally high IgE/IgG ratio in patient BP‐60 (not shown), suggesting that a different phenomenon might be at play in this individual. Despite the fact that BP IgG and IgE recognize the same BP180 epitopes, a statistically significant competition effect could not be measured via ELISA. This is likely due to the fact that IgE concentrations in serum are too low and, therefore, small changes in IgE reactivity cannot be appropriately detected with the ELISA technique employed in this study. Another explanation could be that IgE has higher affinity towards the antigens than IgG and a long and colder incubation, as is the case in the ELISA assay, leads to substitution of part of the IgG molecules, which attach quickly, by IgE. Alternatively, it could be that the affinity for different epitopes masks the effect of the competition for the overlapping ones.

Concerning the distribution of BP‐specific IgG subclasses, we have found that, in our patients, the extent of IgG4 staining along the BMZ is greatly superior to that of IgG1 and IgG3, despite the fact that all these patients deposit complement along this structure. This may imply that complement deposition in BP skin occurs in an IgG‐independent fashion, e.g. via binding to adhesion proteins,^44^ alternative complement pathway components^45^ or even DNA.^46^ Nevertheless, there are several reasons why we believe that IgG1/IgG3 α‐BP antibodies are responsible for complement deposition along the BMZ. Firstly, 10% of our patients did show a clear deposition of IgG1 and IgG3 along the BMZ by DIF. In the other patients, we would assume that the amounts of IgG1 and/or IgG3 were too minute to be detectable by immunofluorescence, but were still present and amplified by complement deposition and activation. This assumption gains support from our finding that BMZ‐specific IgG1, IgG3 and IgG4 are all found in BP sera and from immunofluorescence studies in patients with pemphigoid (herpes) gestationis. In these patients, IgG can barely be detected in perilesional skin, whereas complement deposition is regularly present.^47^, ^48^ *In vitro* experiments showed the capacity of these autoantibodies to fix complement and therefore amplify the immunofluorescence signal.

In our study, the most prominent expression of IgE immunopathology in patients with BP was the occurrence of IgE+ leucocytes, predominantly eosinophils and mast cells, in perilesional skin. In the case of mast cells, this finding was not entirely unexpected.^22^, ^49^ To the best of our knowledge, the presence of IgE on lesional eosinophils has not been reported so far. Our stripping and reconstitution experiments strongly imply that IgE molecules in BP skin are bound via FcεRI on all IgE+ cell populations. This assumption is in agreement with the identification of surface FcεRI on eosinophils in the blood of patients with BP.^32^ All these findings support the hypothesis that IgE accumulates at sites rich in high‐affinity IgE receptors, thus having the potential to be pathogenic by itself.

The major question then remained as to whether cell‐bound IgE in perilesional BP skin could interact with BP antigens and, if so, what the functional consequences of such a liaison would be. For this purpose, we analysed BP skin sections for both the intra‐ and the extracellular domain of BP180. While intracellular BP180 was present only along the BMZ, fragments of the extracellular domain could be found along the BMZ and in the dermis, not only in patients with BP, but also in HCs. This supports and confirms the hypothesis that BP180 shedding by keratinocytes is a naturally occurring phenomenon.^34^, ^50^, ^51^ Most importantly, we observed that, as opposed to the situation in controls, where BP180 fragments were only localized in a random fashion, in patients with BP these were also seen in association with eosinophil‐bound IgE and could be removed by acid stripping. These results complement the observation of IgE‐ or BP180‐covered mast cells in BP skin, found by other investigators.^22^ Although we have not formally excluded the remote possibility of BP180+/IgE+ leucocytes being present in the circulation, our data strongly suggest that IgE and BP180 do form immune complexes in BP skin, conceivably triggering events resulting in tissue inflammation.

To address the functional potential of IgE‐BP180 complexes, we sequentially stimulated FcεRI‐expressing rat basophils with BP sera and BP180 peptides *in vitro* and were able to show that IgE‐BP180 complexes are indeed capable of cross‐linking FcεRI, ultimately leading to degranulation. This finding complements previous data on human circulating basophils and eosinophils.^32^ Degranulation was seen in the two patients with the highest levels of BP180‐specific IgE in ELISA. In other patients, the concentration of BP‐specific serum IgE was probably too low and that of other isotypes too high to allow the delivery of a robust activation signal. This assumption is supported by our observation of a significant increase in degranulation when IgG‐depleted serum vs. nondepleted serum was used.

Without denying or downplaying the importance of an IgG1/IgG3/complement axis in BP development, the results of this study add further support, in a nonexclusive fashion, for the existence of a pathogenetically relevant second pathway, perhaps T helper 2 dependent,^52^, ^53^, ^54^ carried by BP‐specific IgE and, probably, IgG4 antibodies. The factors governing the predominance and interdependence of these two pathways are entirely unknown, as is the temporal sequence in the elicitation and expansion of these axes. While firm evidence is lacking, it is tempting to speculate that an initial IgG response, even if modest and asymptomatic, is perhaps either necessary for or facilitates a second‐line response, dependent on IgE. Some studies show an improvement of BP symptoms and disease markers following treatment with α‐IgE therapy.^55^, ^56^ Nonetheless, well‐controlled clinical trials with molecules interfering with the critical players of both pathways are needed to determine their relative importance in disease elicitation and perpetuation.

In conclusion, by combining data from serum and skin, the present study strongly implies that IgE‐BP180 complexes present in the skin may activate mast cells and eosinophils via FcεRI and, thus, may be involved in the development of certain clinical symptoms typical of BP.

## Supporting information


**Data S1** Supplementary materials and methods (see Fig. S1).

**Fig S1.** Verification of the specificity of the α‐IgE antibody.

**Fig S2.** Patients with bullous pemphigoid (BP) have elevated levels of total circulating IgE.

**Fig S3.** No significant competition, between IgG and IgE, for BP180‐NC16A can be detected by enzyme‐linked immunosorbent assay.

**Fig S4.** Tissue IgE does not appear to correlate with total or specific circulating IgE.

**Fig S5.** Rare case of a patient with bullous pemphigoid with linear deposits of IgE in direct immunofluorescence and indirect immunofluorescence.

**Fig S6.** Eosinophils from patients with bullous pemphigoid express FcεRI.

**Fig S7.** Bullous pemphigoid (BP) perilesional and non‐BP disease skin have significantly more eosinophils than healthy skin; in contrast, all skin samples exhibit similar amounts of mast cells and BP180 dermal fragments.

**Fig S8.** IgG autoantibodies shift from a balanced distribution in serum to an IgG4 dominant profile in skin.

**Fig S9.** BP180 is found along the basement membrane zone and in the dermis.

**Fig S10.** No significant differences are found between cohorts of healthy controls and patients with bullous pemphigoid for the degranulation of basophils.

**Video S1** Author video.
