# Supplementary material for: IgE autoreactivity in bullous pemphigoid: eosinophils and mast cells as major targets of pathogenic immune reactants
Source: Br J Dermatol. 2017 Nov 28;177(6):1644–53. doi: 10.1111/bjd.15924 (PMC5814899; doi:10.1111/bjd.15924)
Supplement: Supplementary file 1 — Data S1 Supplementary materials and methods (see Fig. S1). [file BJD-177-1644-s001.docx]

SUPPLEMENTARY MATERIALS AND METHODS

**Sera and skin samples**

BP sera (n=19) were collected from patients who voluntarily signed an informed consent form. Sera from healthy donors (n=18) were collected from volunteers who also gave written permission. All sera were aliquoted and stored at -20°C or -80°C.

Skin biopsies from BP patients (n=33) and those with non-BP skin conditions (n=8) were collected for routine diagnostic purposes, embedded in optimal cutting temperature (OCT) medium (Tissue-Plus, Scigen Scientific Gardena, CA), snap-frozen in liquid nitrogen and stored at -80°C in the Department of Dermatology of Vienna’s General Hospital. Patients were informed in writing of the use of routine biopsies for scientific purposes. Non-BP controls comprised patients diagnosed with: linear IgA disease (n=1), vasculitis (n=1), urticaria (n=1), epidermolysis bullosa acquisita (n=2), pemphigus vulgaris (n=2) and dermatitis herpetiformis (n=1). Normal human skin (n=5) was obtained from patients undergoing abdominoplastic surgery.

This study was approved by the Ethics Committee of the Medical University of Vienna (EK1645/2014) and conducted according to the Declaration of Helsinki.

**IgG subclasses**

IgG subclasses in sera and skin were determined by indirect immunohistochemistry on monkey esophagus slides (Inova Diagnostics, San Diego, CA) or by direct immunohistochemistry, respectively. Detailed information on the antibodies used can be found in Supplementary table 1.

**Assessment of IgG cross-reactivity by α-IgE antibodies**

Approximatelly 3 x 10^5^ poly(styrene/divinylbenzene) beads (Bangs Laboratories, Fishers, IN) were incubated with different dilutions of human IgG (Abcam, Cambridge, United Kingdom) or IgE (Abcam). The excess immunoglobulins were washed off and the beads incubated with either rabbit α-human IgG (Dako, Glostrup, Denmark), or α-human IgE (see supplementary table 1). Fluorescent beads were detected in a FACS Calibur device (BD Biosciences, San Jose, CA).

**α-BP180 and α-BP-230 IgE ELISAs**

NC16A and BP230 peptide-covered ELISA plates were obtained commercially (Mesacup, MBL, Nagoya, Japan) and adapted for IgE detection. ELISA plates were left incubating with 50 µL of BP or control sera, diluted 1:25 in phosphate buffer saline (PBS), over-night at 4°C. Details on the detection antibodies used can be found in the Supplementary Table 1. The α-IgE antibody used did not show cross-reactivity with IgG (Supplementary figure 3). Wash buffer, tetramethylbenzidine (TMB) substrate and stop solution were provided in the kit. Absorbance was read at 450 nm with correction at 620 nm.

**IgG depletion**

Depletion of IgG from BP and normal sera was performed using protein G SpinTrap columns (GE Healthcare, Buckinghamshire, United Kingdom). IgG IIF on monkey esophagus was performed to determine the success of IgG depletion. Negative sera at a dilution of 1:10 were considered as thoroughly depleted of IgG.

**IgG vs IgE competitive ELISA**

IgG was isolated from BP serum using protein G SpinTrap colums (GE Healthcare, Buckinghamshire, United Kingdom). IgG reactivity against BP proteins was tested by IIF on monkey esophagus slides. IgE ELISAs were run as described in Materials and Methods, except 10-1000 μg/mL of total purified BMZ-reactive or non-reactive IgG were added to the ELISA plates for 1 hour at room temperature, prior to incubation with BP serum.

**Uncovering of the major BP-180 epitopes**

Overlapping peptides spanning the NC16A and C-terminal domains of BP180 (see Supplementary Table 2 for peptides’ sequences) were kindly provided by Marwa Mostageer and Oskar Smrzka at Affiris AG (Vienna, Austria) as bovine serum albumin (BSA)-conjugated fragments. These were plated over-night at 4°C onto MediSorp 96-well plates (Thermo Scientific, Waltham, MA) and the unbound peptides washed with 0.1% TWEEN-20 (Sigma-Aldrich, St. Louis, MO) in PBS. After a 1 hour blocking step with 1% BSA (Sigma-Aldrich) in PBS, BP or control sera were diluted in 0.1 % BSA / 0.1 % TWEEN-20 / PBS to 1:200 (for IgG detection) or 1:20 (for IgE detection) and left incubating for 1 hour at room temperature or over-night at 4°C, respectively. α-human IgG or IgE were added for 1 or 2 hours, respectively, at room temperature (see Supplementary Table 1 for antibody details). After a final wash, TMB (eBioscience, San Diego, CA) was left reacting for 20 minutes, after which a 1 N solution of phosphoric acid (Sigma-Aldrich) was added. The colorimetric result was measured at 450 nm with correction at 620 nm. An FcεRI fragment was used as an irrelevant peptide and any signal 2-fold higher than that of the negative control was considered positive.

**Stainings, quantification and stripping of skin-bound IgE**

Cryopreserved skin biopsies were cut to 5 µm sections, placed onto Superfrost Ultra Plus glass slides (Thermo Scientific) and fixed with cold acetone (Sigma-Aldrich) for 10 minutes. To reduce background, a pre-incubation with a 2 % solution of BSA, mouse, goat or rabbit serum in PBS or with a 1:20 dilution of Fc receptor blocking reagent (BD Biosciences, San Jose, CA), for 20 minutes at room temperature, was performed. Slides were then incubated with primary antibodies, washed 3 times for 5 minutes with PBS and, when appropriate, incubated with secondary antibodies, followed by another washing step. Finally, 4',6-diamidino-2-phenylindole (DAPI) (Thermo Scientific) was added to stain the nuclei and the slides washed and covered with PermaFluor (Thermo Scientific). Detailed information on the commercial antibodies used is provided in the Supplementary Table 1. Mouse α-LAD-1 was a generous gift from Peter Marinkovich at the Stanford University School of Medicine (Stanford, CA)^1^ and α-FcεRI blocking antibody (15_1)^2^ was kindly provided by Jean-Pierre Kinet from Harvard Medical School (Boston, MA).

IgE stripping was performed on acetone-fixed sections, using a glycine buffer (50 mM glycine, 85 mM NaCL, 5 mM KCl, 10 mM EDTA, pH 2.8, Sigma-Aldrich) as previously described^3^. Post-stripped sections were incubated either with human IgE (Abcam, Cambridge, United Kingdom) alone or blocked first for 2 hours at room temperature with 10 µg/mL of α-FcεRI (15_1) or α-CD23 (BD Biosciences) before adding IgE.

Images were obtained either with an Axiovert 200 M laser scan microscope (LSM) 410 (Carl Zeiss, Jena, Germany) or with an Axio Observer Z1 microscope (Carl Zeiss) operated by a TissueFAXS imaging system (TissueGnostics, Vienna, Austria). Quantification was performed with TissueQuest software (TissueGnostics), using isotypes for each patient-staining pair as negative controls.

**IgE-mediated basophil degranulation**

Human FcεRI-expressing rat basophil leukemia cells (RBL) were a gift from Ryosuke Nakamura (National Institute of Health Sciences, Tokyo, Japan)^4^, and the experiments were performed as previously described^5^, with minor modifications. Briefly, cells were plated in 96-well plates, incubated over-night at 37°C with a 1:10 dilution of BP sera in Minimum Essential Media (MEM, Invitrogen, Waltham, MA) and stimulated for 1 hour at 37°C with different allergen dilutions in Tyrode’s Buffer (137 mM NaCl, 2.7 mM KCl, 0.5 mM MgCl_2_, 1.8 mM CaCl_2_, 0.4 mM NaH_2_PO_4_, 5.6 mM d-glucose, 12 mM NaHCO_3_, 10 mM N-2-hydroxyethylpiperazine-N′-2-ethanesulfonic acid and 0.1% BSA, pH 7.2, Sigma-Aldrich), containing 50 % D_2_O (Sigma-Aldrich). The allergens were selected for having strong or low reactivity with BP sera (see methods above and Figure 1c). Chimeric human IgE, kindly provided by Rudolf Valenta at the Medical University of Vienna (Vienna, Austria)^6^, and α-human IgE (Beckman Coulter, Brea, CA) were used as a positive control, whereas buffer alone was used as a negative control. Degranulation was measured via the detection of β-hexosaminidases using 4-methylumbelliferyl phosphate (Sigma-Aldrich). Glycine buffer (0.2 M glycine and 0.2 M NaCl, pH 10.7, Sigma-Aldrich) was added after 1 hour to stop the chemical process. The reaction product has excitation and emission wave-lengths at 360 and 449 nm, respectively. Results were expressed as a percentage of total β-hexosaminidase release, obtained by lysing the cells with 10 % Triton-X-100 (Sigma-Aldrich).

Supplementary table 1: Commercial primary and secondary antibodies

|  | Target | Label | Host | Dilution | Manufacturer |
| --- | --- | --- | --- | --- | --- |
| α-BP180/230 IgE ELISA | | | | | |
|  | Human IgE | Biotin | Mouse | 1:100 | eBioscience (San Diego, CA) |
| BP180 dominant epitopes ELISA | | | | | |
|  | Human IgG | HRP | Mouse | 1:1000 | Jackson ImmunoResearch (West Grove, PA) |
|  | Human IgE | Biotin | Mouse | 1:100 | eBioscience |
| Immunofluorescence stainings | | | | | |
|  | MBP | Z*AF568 | Mouse | 1:200 | Fitzgerald (North Acton, MA) |
|  | ECP | AF555 | Rabbit | 1:500 | Bioss (Woburn, MA) |
|  | CD117 | APC | Mouse | 1:50 | Beckman Coulter (Brea, CA) |
|  | Collagen XVII  (aa 1-100) | - | Rabbit | 1:250 | Abcam (Cambridge, United Kingdom) |
|  | Human IgE | - | Mouse | 1:1000 | Southern Biotech (Birmingham, AL) |
|  | Human IgE | FITC | Goat | 1:100 | Invitrogen (Carlsbad, CA) |
|  | Human IgE | - | Goat | 1:100 | Abcam |
|  | Human IgE | - | Mouse | 1:100 | eBioscience |
| IgG Subclasses | | | | | |
|  | Human IgG1 | HRP | Mouse | 1:200 | Molecular Probes (Eugene, OR) |
|  | Human IgG2 | HRP | Mouse | 1:200 | Life Technologies (Carlsbad, CA) |
|  | Human IgG3 | HRP | Mouse | 1:200 | Life Technologies |
|  | Human IgG4 | HRP | Mouse | 1:200 | Southern Biotech |
| Isotype controls | | | | | |
|  | - | - | Mouse | Variable | Sigma-Aldrich (St. Louis, MO) |
|  | - | Z*AF568 | Mouse | 1:2000 | Sigma-Aldrich |
|  | - | AF555 | Rabbit | 1:500 | Bioss |
|  | - | APC | Mouse | 1:400 | BD Biosciences (San Jose, CA) |
|  | - | - | Rabbit | 1:1000 | Dako (Glostrup, Denmark) |
|  | - | FITC | Goat | 1:100 | Cedarlane (Burlington, Canada) |
|  | - | - | Goat | 1:5000 | Life Technologies |
| Secondary Antibodies | | | | | |
|  | Biotin | HRP | - | 1:1000 | Perkin-Elmer (Waltham, MA) |
|  | Goat IgG | AF488 | Rabbit | 1:400 | Molecular Probes |
|  | Mouse IgG | AF546 | Goat | 1:400 | Molecular Probes |
|  | Mouse IgG | FITC | Goat | 1:400 | Molecular Probes |
|  | Rabbit IgG | FITC | Goat | 1:1000 | Abcam |

Abbreviations: MBP: major basic protein; ECP: eosinophil cationic protein; HRP: horseradish peroxidase; AF: Alexa Fluor; APC: allophycocyanin; FITC: fluorescein isothiocyanate; Z*: zenon-labelled antibody (Thermo Scientific, Waltham, MA); Zenon labelling was performed according to the manufacturer’s instructions.

**Supplementary table 2: Amino acid sequence for BP180 NC16A- and C-terminal-spanning peptides**

| Region | Peptide’s name | Sequence |
| --- | --- | --- |
| NC16A | BP180-489 | CAEEVRKLKARVDE |
|  | BP180-496 | CKARVDELERIRRS |
|  | BP180-503 | CERIRRSILPYGDS |
|  | BP180-510 | CLPYGDSMDRIEKD |
|  | BP180-517 | CDRIEKDRLQGMAP |
|  | BP180-524 | CLQGMAPAAGADLD |
|  | BP180-531 | CAGADLDKIGLHSD |
|  | BP180-538 | CIGLHSDSQEELWM |
|  | BP180-545 | CQEELWMFVRKKLM |
|  | BP180-550 | CMFVRKKLMMEQEN |
| C-terminal | BP180-1331 | CAGDRGPYGTDIGP |
|  | BP180-1338 | CGTDIGPGGGYGAA |
|  | BP180-1345 | CGGYGAAAEGGMYA |
|  | BP180-1352 | CEGGMYAGNGGLLG |
|  | BP180-1359 | CNGGLLGADFAGDL |
|  | BP180-1366 | CDFAGDLDYNELAV |
|  | BP180-1373 | CYNELAVRVSESMQ |
|  | BP180-1381 | CSESMQRQGLLQGM |
|  | BP180-1391 | CQGMAYTVQGPPGQ |

**REFERENCES**

1. Marinkovich PM, Taylor TB, Keene DR, Burgeson RE, Zone JJ. LAD-1, the Linear IgA Bullous Dermatosis Autoantigen, Is a Novel 120-kDa Anchoring Filament Protein Synthesized by Epidermal Cells. *J Invest Dermatol*. 1996;106(4):734-738. doi:http://dx.doi.org/10.1111/1523-1747.ep12345782.

2. Wang B, Rieger A, Kilgus O, et al. Epidermal Langerhans cells from normal human skin bind monomeric IgE via Fc epsilon RI. *J Exp Med*. 1992;175(5):1353-1365.

3. Klubal R, Osterhoff B, Wang B, Kinet J-P, Maurer D, Stingl G. The High-Affinity Receptor for IgE Is the Predominant IgE-Binding Structure in Lesional Skin of Atopic Dermatitis Patients. *J Invest Dermatol*. 1997;108(3):336-342. doi:http://dx.doi.org/10.1111/1523-1747.ep12286482.

4. Takagi K, Nakamura R, Teshima R, Sawada J. Application of human Fc epsilon RI alpha-chain-transfected RBL-2H3 cells for estimation of active serum IgE. *Biol Pharm Bull*. 2003;26(2):252-255.

5. Gieras A, Focke-Tejkl M, Ball T, et al. Molecular determinants of allergen-induced effector cell degranulation. *J Allergy Clin Immunol*. 2007;119(2):384-390. doi:http://dx.doi.org/10.1016/j.jaci.2006.09.034.

6. Laffer S, Hogbom E, Roux KH, et al. A molecular model of type I allergy: Identification and characterization of a nonanaphylactic anti-human IgE antibody fragment that blocks the IgE-FcϵRI interaction and reacts with receptor-bound IgE. *J Allergy Clin Immunol*. 2001;108(3):409-416. doi:http://dx.doi.org/10.1067/mai.2001.117593.
