# Supplementary figures and images for: IgE autoreactivity in bullous pemphigoid: eosinophils and mast cells as major targets of pathogenic immune reactants
Source: Br J Dermatol. 2017 Nov 28;177(6):1644–53. doi: 10.1111/bjd.15924 (PMC5814899; doi:10.1111/bjd.15924)

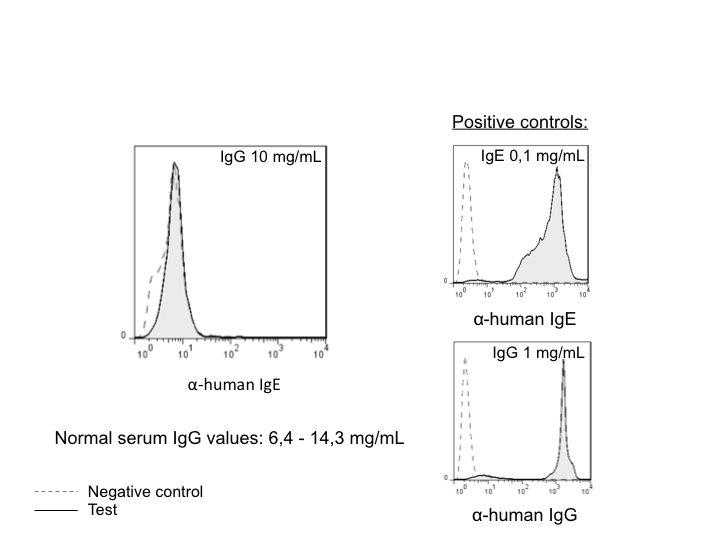

Supplement: Supplementary file 2 — Fig S1. Verification of the specificity of the α‐IgE antibody. [file BJD-177-1644-s002.tiff]

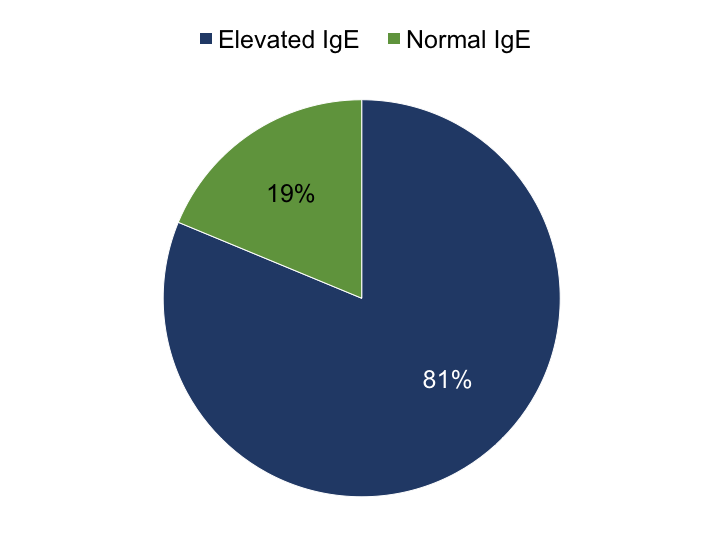

Supplement: Supplementary file 3 — Fig S2. Patients with bullous pemphigoid (BP) have elevated levels of total circulating IgE. [file BJD-177-1644-s003.tiff]

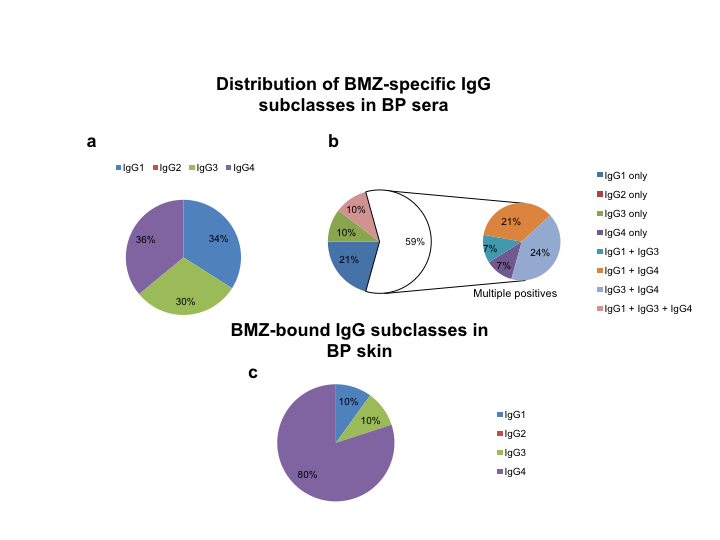

Supplement: Supplementary file 9 — Fig S8. IgG autoantibodies shift from a balanced distribution in serum to an IgG4 dominant profile in skin. [file BJD-177-1644-s009.tiff]
